# Willingness of adults in the United States to have a multicancer detection test

**DOI:** 10.1007/s10552-025-02014-2

**Published:** 2025-05-31

**Authors:** Paul L. Reiter, Mira L. Katz

**Affiliations:** 1 College of Public Health, The Ohio State University, Columbus, OH USA; 2 Comprehensive Cancer Center, The Ohio State University, Columbus, OH USA

**Keywords:** Cancer, Cancer screening, Multicancer detection, Willingness

## Abstract

**Purpose:**

Multicancer detection (MCD) testing is an emerging approach with the potential to improve the cancer screening and early detection landscape. As the clinical utility of MCD testing is further determined, it is important to examine patients’ willingness to have an MCD test.

**Methods:**

We conducted an online survey in September 2024 with a national sample of adults aged 45–80 from the United States (*n* = 1,043). Multivariable logistic regression identified correlates of participants’ willingness to have an MCD test.

**Results:**

About three-fourths of participants (75.2%) indicated they were willing to have an MCD test if it was free or covered by health insurance, while only 16.9% were willing if the test cost $1,000 out of pocket. Participants were more willing to have a free MCD test if they had at least a college degree (odds ratio [OR] = 2.48, 95% confidence interval [CI] 1.12–5.48), had some form of health insurance (OR = 3.40, 95% CI 1.84–6.27), or had a routine medical check-up within the last year (OR = 1.62, 95% CI 1.01–2.59). The most commonly endorsed factors that would matter in participants’ hypothetical MCD testing decisions were cost or health insurance coverage (60.7%), how well the test works (50.6%), and if a doctor recommended having a test (47.6%).

**Conclusions:**

Most adults are willing to have an MCD test, if it is free or covered by health insurance. Findings can help guide programs about MCD testing for both patients and healthcare providers that are becoming increasingly necessary as MCD testing continues to be examined as a screening strategy.

## Introduction

Cancer screening is an effective and cost-effective approach for detecting cancer at early stages and reducing cancer mortality [1–4]. However, only a few cancer types (i.e., breast, cervical, colorectal, and lung cancers) have screening tests that are currently recommended for routine use by the United States Preventive Services Taskforce (USPSTF) [5–7]. This means that routine screening is not available and/or recommended for cancer types that account for about 70% of all cancer deaths [8]. Furthermore, the cancer screening tests that are currently recommended for routine use continue to be underutilized in the US and remain below national screening goals [9]. Recent data suggest that about 25% of age-eligible women in the US are not within current screening guidelines for cervical cancer and breast cancer, and more than 30% of age-eligible adults are not within current screening guidelines for colorectal cancer [10].

A potential new screening and early detection approach is multicancer detection (MCD) testing. MCD testing involves a blood test that screens for cancer by detecting biomarkers that are either released by or induced by cancer cells. Multiple MCD tests have been developed, with the different tests varying on the number and types of cancer able to be detected (e.g., the number of detectable cancer types ranges from fewer than five to more than 50) [11]. The performance of the different tests also varies, with sensitivities for all detectable cancers and across all stages ranging from 27 to 95% at specificities of 95–99% [11–13]. It is worth noting that performance metrics also vary across individual cancer types [11–13]. Randomized trials are underway to provide further information on the performance and clinical utility of MCD testing among asymptomatic individuals [11, 12]. Currently, MCD tests may be introduced into clinical care as laboratory-developed tests (LDTs) and are therefore subject to regulations and regulatory decisions involving LDTs [12]. None of the MCD tests have yet received approval by the US Food and Drug Administration (FDA), but some have already become commercially available [14].

As research continues to examine the potential of MCD testing in clinical settings, it is important to also begin understanding people’s perspectives about MCD testing and their willingness to have an MCD test. However, only a few studies thus far have examined willingness to have an MCD test among adults in the US [15–18]. Two of these studies were quantitative and found that a majority of participants would be willing to have an MCD test [15, 16], which is similar to studies in the United Kingdom that also reported high levels of interest in MCD testing among participants [19, 20]. Given the lack of behavioral research on MCD testing, additional efforts are needed to examine topics that are highly relevant at this early point for MCD testing, including people’s awareness of MCD testing, willingness to have an MCD test, and factors that would affect their decisions about whether or not to have an MCD test.

The current study examined these topics among a national sample of adults in the US. In doing so, we provide further evidence on people’s willingness to have an MCD test and new insight into awareness of MCD testing and factors that would affect MCD testing decisions. Results will be able to help guide the development and content of future programs about MCD testing for patients and healthcare providers (e.g., education programs, communication training) that will likely become increasingly necessary.

## Materials and methods

### Study design

We conducted a cross-sectional online survey about health topics among adults in the US in September 2024. One of the topics addressed on the survey was MCD testing. Eligibility criteria for the survey included currently living in the US and being 45–80 years of age, which is similar to the age range of recent and current clinical studies involving MCD testing [11, 21, 22]. All participants were recruited via an online survey panel accessed through the research company SSRS (Glen Mills, PA). Members of the panel are invited by the research company to complete self-administered online surveys on a regular basis in exchange for incentives.

We recruited a convenience sample of participants from this online panel. Panel members who were potentially eligible received an email invitation from SSRS to participate. The invitation included a weblink to a brief screener survey to determine study eligibility. Panel members who were confirmed eligible were then asked to provide informed consent prior to completing their study survey. A total of 1,043 adults from 46 states participated in our study. The mean duration of survey completion was just over 19 min, and participants received standard incentives from SSRS for completing the survey. The Institutional Review Board at The Ohio State University determined this study was exempt from review.

### Measures

#### MCD testing

We developed survey items based on recent studies examining MCD testing and our past research on emerging health behaviors [15, 20, 23–25]. We refined the survey and its items via an iterative process prior to data collection. At the start of the survey section on MCD testing, participants were provided with an informative statement about MCD testing (the survey included the acronym MCED testing instead of MCD testing):

“A new type of cancer screening test, called a multi-cancer early detection test or MCED test, is being developed by researchers. An MCED test can screen for multiple types of cancer at the same time. For this test, a person has a blood draw taken from their arm by a doctor or other health care provider. The blood sample is then tested for potential signs of cancer by a lab.

Several MCED tests are being developed, but none have yet been approved for routine use. Some of the tests can help screen for more than 50 different types of cancer at the same time. This includes some types of cancer that currently have no other screening test available (e.g., stomach cancer).

If an MCED test result is positive (i.e., abnormal), additional tests are then done to confirm if a person has cancer.”

The survey then asked participants if they were aware of MCD testing prior to taking the survey. For those participants who indicated they were aware, a follow-up question examined the sources where participants had heard or seen information about MCD testing. Responses options included newspaper, magazine, radio, television, social media, internet but not social media, family members or friends, and doctor or other health care provider. The survey allowed participants to indicate multiple sources.

The survey then assessed our primary outcome of participants’ willingness to have an MCD test with two items. The first item was, “How willing would you be to get an MCED test to screen for cancer if it was free or covered by health insurance?” The second item was, “How willing would you be to get an MCED test to screen for cancer if it cost $1,000 out of pocket? This would be from your own money, not paid for by insurance.” At the time of data collection, the price of two MCD tests that can detect a range of cancer types and that were already commercially available was about $1,000 each [11]. The ordering of the two willingness items was randomized, and response options for both items were “definitely not willing,” “probably not willing,” “not sure,” “probably willing,” and “definitely willing” for both items (coded 1–5). For regression analyses (described further below), we categorized each participant as either “willing” (definitely or probably willing) or “not willing” (all other responses) to have an MCD test if it was free. We examined free MCD testing in regression analyses, as most health insurance plans would likely cover the costs of MCD testing if it becomes recommended by the USPSTF [26].

The survey then assessed our secondary outcome of factors that would matter in participants’ hypothetical decisions about whether or not to have an MCD test. This involved a survey activity that indicated to participants, “Imagine you are considering whether or not to get an MCED test. Several factors may affect this decision. For each of the following factors, indicate if it would affect your decision to get an MCED test “A great deal”, “A little bit”, or “Not at all”.” The survey then presented 10 factors (e.g., cost and health insurance coverage, how well the test works, if a doctor recommended to have the test) that we believed would be relevant to people’s decisions about MCD testing. Factors were presented one at a time to participants, and the ordering of factors was randomized. For analyses, we dichotomized responses for each factor as either “a great deal” or “a little bit or not at all.”

#### Demographic and health-related characteristics

The survey assessed a range of demographic and health-related characteristics (Table 1). We used the 2010 Rural–Urban Commuting Area (RUCA) codes [27] to classify each participant as living in an “metropolitan” (RUCA codes of 1–3) or “non-metropolitan” (RUCA codes of 4–10) area. We examined participants’ health insurance status, if they had a routine medical check-up within the last year, and health literacy. Health literacy was examined using the Single Item Literacy Screener that can categorize participants as having either “adequate” or “limited” health literacy [28].

**Table 1:** Demographic and health-related characteristics of participants (*n* = 1,043)

	*n* (weighted %)
Demographic characteristics	
Sex	
Female	516 (49.9)
Male	527 (50.1)
Age (years)	
45–54	354 (30.1)
55–64	316 (31.3)
65–80	373 (38.6)
Race/ethnicity	
Non-Hispanic white	543 (60.0)
Non-Hispanic black	64 (12.3)
Hispanic	410 (19.9)
Non-Hispanic other	26 (7.8)
Relationship status	
Never married	225 (17.2)
Married/civil union or living with partner	469 (56.2)
Divorced, separated, or widowed	349 (26.6)
Education level	
Less than high school degree	106 (11.2)
High school degree	344 (40.2)
Some college	322 (21.0)
College degree or more	271 (27.6)
Household income	
Less than $50,000	658 (58.2)
$50,000 to $89,999	242 (23.8)
$90,000 or more	143 (18.0)
Political party affiliation	
Democrat	406 (32.6)
Independent/other	300 (34.1)
Republican	337 (33.3)
Religiosity	
Not at all or slightly important	294 (30.2)
Fairly, very, or extremely important	749 (69.8)
Sexual identity	
Straight/heterosexual	976 (93.5)
Some other identity	67 (6.5)
Metropolitan status	
Metropolitan	876 (82.1)
Non-metropolitan	167 (17.9)
Region of residence	
Northeast	186 (17.3)
Midwest	176 (20.8)
South	438 (39.0)
West	243 (22.9)
Health-related characteristics	
Health insurance	
No	98 (9.4)
Yes	945 (90.6)
Routine medical check-up within the last year	
No	266 (24.2)
Yes	777 (75.8)
History of any type of cancer	
No	883 (84.8)
Yes	160 (15.2)
Health literacy	
Limited	141 (13.9)
Adequate	902 (86.1)

### Data analysis

Our primary outcome was participants’ willingness to have an MCD test, and we compared willingness to get an MCD if it was free or cost $1,000 out of pocket using a paired *t* test. We then used logistic regression to identify demographic and health-related characteristics correlated with willingness to have an MCD test if it was free. For logistic regression, we entered all variables with *p* < 0.10 in bivariate analyses into a multivariable model. These models produced odds ratios (ORs) and 95% confidence intervals (CIs). Lastly, we examined our secondary outcome of factors that would matter in participants’ hypothetical decisions about whether or not to have an MCD test. Since some participants did not complete the survey activity involving these factors, we first used chi-square tests to compare those who completed it to those who did not on demographic and health-related characteristics. Among participants who completed the survey activity, we calculated descriptive statistics for each of the 10 factors. We then used chi-square tests to compare participants who had a routine medical check-up within the last year to those who did not on each factor, since recent preventive healthcare utilization may affect decision-making factors.

All analyses were conducted using IBM SPSS version 29 (IBM Corp., Armonk, NY), and data were weighted to represent the 45-to-80-year-old population of the US. We report unweighted frequencies, but all other reported statistics are weighted. Statistical tests were two-tailed with a critical alpha of 0.05.

## Results

### Participant characteristics

About half of participants were female (49.9%), and the age distribution included 30.1% who were aged 45–54, 31.3% aged 55–64, and 38.6% aged 65–80 (Table 1). Forty percent of participants indicated a minoritized racial/ethnic identity. About half of participants were married or living with a partner (56.2%), had at least some college education (48.6%), and reported a household income of less than $50,000 (58.2%). Political party affiliation included 32.6% of participants indicating they were a Democrat, 33.3% indicating they were a Republican, and 34.1% indicating they were an Independent or some other affiliation. Most participants lived in a metropolitan area (82.1%), had some form of health insurance (90.6%), and reported having a routine medical check-up within the last year (75.8%).

### Awareness and willingness for MCD testing

Overall, 7.5% (89/1043) of participants were aware of MCD testing prior to the survey. Among participants who indicated they were aware, the most common sources where they had heard or seen information about MCD testing were social media (32.4%), television (29.3%), doctor or other healthcare provider (27.4%), family members or friends (22.3%), the internet but not social media (21.3%), and a newspaper (17.7%). All other sources were indicated by fewer than 15% of participants.

**Fig. 1:**
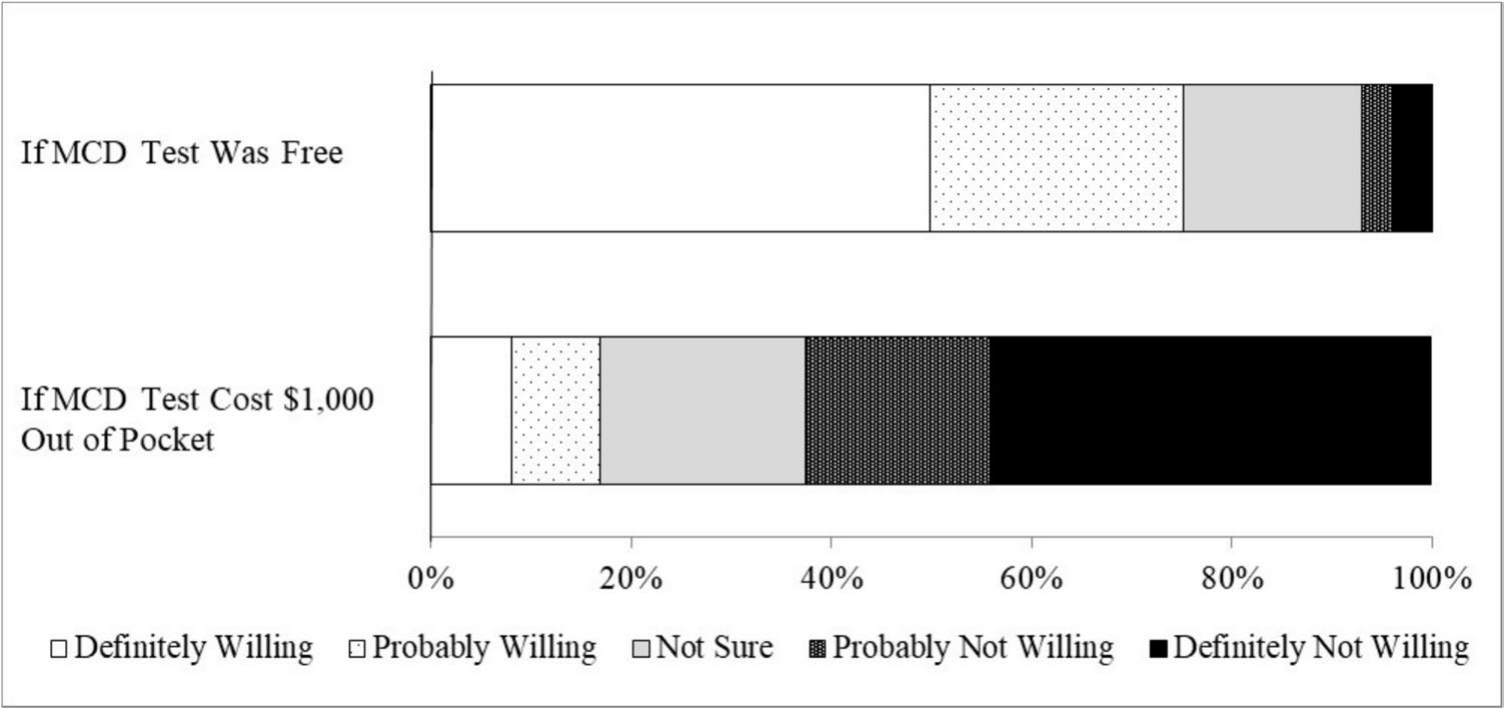
Willingness to have a multicancer detection (MCD) test (*n* = 1,043)

Participants reported higher levels of willingness to have an MCD test if the test was free (mean = 4.14, standard error [SE] = 0.04) than if the test cost $1,000 out of pocket (mean = 2.18, SE = 0.05) (*p* < 0.001). This included 75.2% definitely or probably willing to have an MCD test if the test was free compared to only 16.9% if the test cost $1,000 out of pocket (Fig. 1). Conversely, only 7.1% of participants were definitely or probably not willing to have an MCD test if the test was free, whereas 62.5% of participants were definitely or probably not willing if the test cost $1,000 out of pocket.

In multivariable analyses (Table 2), participants were more likely to be willing to have a free MCD test if they had at least a college degree (OR = 2.48, 95% CI 1.12–5.48), had some form of health insurance (OR = 3.40, 95% CI 1.84–6.27), or reported having a routine medical check-up within the last year (OR = 1.62, 95% CI 1.01–2.59). Participants who were non-Hispanic black were less willing to have a free MCD test compared to participants who were non-Hispanic white (OR = 0.42, 95% CI 0.20–0.87).

**Table 2:** Correlates of willingness to have a multicancer detection (MCD) test (*n* = 1,043)

	*n*	Willing to have free MCD test (weighted %)	Not willing to have free MCD test (weighted %)	Bivariate OR (95% CI)	Multivariable OR (95% CI)
Demographic characteristics					
Sex					
Female	516	392 (72.8)	124 (27.2)	Ref	–
Male	527	422 (77.5)	105 (22.5)	1.29 (0.87–1.91)	–
Age (years)					
45–54	354	262 (71.8)	92 (28.2)	Ref	–
55–64	316	244 (73.9)	72 (26.1)	1.14 (0.69–1.80)	–
65–80	373	308 (78.8)	65 (21.2)	1.46 (0.91–2.34)	–
Race/ethnicity					
Non-Hispanic white	543	427 (77.4)	116 (22.6)	Ref	Ref
Non-Hispanic black	64	41 (61.6)	23 (38.4)	0.47 (0.25–0.88)**	0.42 (0.20–0.87)**
Hispanic	410	329 (80.8)	81 (19.2)	1.23 (0.83–1.80)	1.17 (0.77–1.79)
Non-Hispanic other	26	17 (64.8)	9 (35.2)	0.53 (0.22–1.33)	0.53 (0.22–1.32)
Relationship status					
Never married	225	162 (66.2)	63 (33.8)	Ref	Ref
Married/civil union or living with partner	469	377 (77.9)	92 (22.1)	1.80 (1.08–3.00)**	1.23 (0.68–2.23)
Divorced, separated, or widowed	349	275 (75.1)	74 (24.9)	1.54 (0.89–2.66)	1.31 (0.73–2.36)
Education level					
Less than high school degree	106	71 (66.6)	35 (33.4)	Ref	Ref
High school degree	344	255 (71.3)	89 (28.7)	1.24 (0.69–2.25)	1.12 (0.59–2.12)
Some college	322	253 (72.8)	69 (27.2)	1.34 (0.72–2.52)	1.14 (0.58–2.23)
College degree or more	271	235 (86.1)	36 (13.9)	3.10 (1.56–6.18)**	2.48 (1.12–5.48)**
Household income					
Less than $50,000	658	496 (71.3)	162 (28.7)	Ref	Ref
$50,000 to $89,999	242	205 (80.8)	37 (19.2)	1.69 (1.00–2.87)*	1.12 (0.65–1.94)
$90,000 or more	143	113 (80.1)	30 (19.9)	1.62 (0.94–2.78)*	1.02 (0.53–1.96)
Political party affiliation					
Democrat	406	330 (79.1)	76 (20.9)	Ref	Ref
Independent/other	300	213 (68.2)	87 (31.8)	0.57 (0.35–0.91)**	0.62 (0.37–1.03)*
Republican	337	271 (78.4)	66 (21.6)	0.96 (0.58–1.57)	1.03 (0.60–1.78)
Religiosity					
Not at all or slightly important	294	227 (75.8)	67 (24.2)	Ref	–
Fairly, very, or extremely important	749	587 (74.9)	162 (25.1)	0.95 (0.62–1.47)	–
Sexual identity					
Straight or heterosexual	976	765 (75.0)	211 (25.0)	Ref	–
Some other identity	67	49 (76.8)	18 (23.2)	1.10 (0.54–2.27)	–
Metropolitan status					
Metropolitan	876	677 (74.8)	199 (25.2)	Ref	–
Non-metropolitan	167	137 (77.0)	30 (23.0)	1.13 (0.67–1.91)	–
Region of residence					
Northeast	186	145 (79.2)	41 (20.8)	Ref	–
Midwest	176	140 (73.5)	36 (26.5)	0.73 (0.38–1.40)	–
South	438	335 (73.3)	103 (26.7)	0.72 (0.41–1.26)	–
West	243	194 (76.9)	49 (23.1)	0.88 (0.46–1.65)	–
Health-related characteristics					
Health insurance					
No	98	45 (46.2)	53 (53.8)	Ref	Ref
Yes	945	769 (78.2)	176 (21.8)	4.18 (2.33–7.48)***	3.40 (1.84–6.27)***
Routine medical check-up within the last year					
No	266	176 (63.6)	90 (36.4)	Ref	Ref
Yes	777	638 (78.9)	139 (21.1)	2.14 (1.40–3.27)***	1.62 (1.01–2.59)**
History of any cancer type					
No	883	682 (73.5)	201 (26.5)	Ref	Ref
Yes	160	132 (84.4)	28 (15.6)	1.95 (1.14–3.36)**	1.55 (0.85–2.80)
Health literacy					
Limited	141	105 (71.4)	36 (28.6)	Ref	–
Adequacy	902	709 (75.8)	193 (24.2)	1.25 (0.73–2.16)	–

Multivariable model included all variables with *p* < 0*.*10 in bivariate models. Dashes (–) indicate that variable was not included in the multivariable model

*OR* odds ratio, *CI* confidence interval, *Ref* reference group

*p < 0.10; **p < 0.05; ***p < 0.001

### Factors in MCD testing decisions

A total of 841 participants completed the survey activity that identified factors that would matter in their hypothetical decisions about whether or not to have an MCD test. There were no differences between participants who completed this survey activity and those who did not on demographic and health-related characteristics (all *p* > 0.05). The most commonly endorsed factors that would matter a great deal in participants’ MCD testing decisions were (Table 3): cost or health insurance coverage (60.7%), how well the test works (50.6%), if a doctor recommended having the test (47.6%), if test results indicate the site of cancer a person might have (43.5%), the number of cancer types the test can detect (36.0%), their health history (29.0%), and their age (25.5%). Fewer participants endorsed anxiety about the test and awaiting test results (20.1%), concerns about being able to understand test results (16.9%), and the opinions of family and friends (13.9%).

**Table 3:** Factors that would matter in participants’ hypothetical decisions about whether or not to have a multicancer detection (MCD) test

	Overall (*n* = 841)^a^	Routine medical check-up within the last year (*n* = 624)	No routine medical check-up within the last year (*n* = 217)	*p*-Value
Cost and health insurance coverage	495 (60.7)	380 (61.7)	115 (57.9)	0.471
How well the test works	394 (50.6)	304 (53.2)	90 (43.0)	0.059
If a doctor recommended to have the test	401 (47.6)	329 (54.2)	72 (28.1)	< 0.001
If test results indicate the site of cancer a person might have	369 (43.5)	277 (44.5)	92 (40.4)	0.441
Number of cancer types the test can detect	310 (36.0)	236 (37.3)	74 (32.2)	0.317
Health history	246 (29.0)	188 (29.4)	58 (27.8)	0.746
Age	217 (25.5)	171 (25.8)	46 (24.7)	0.807
Anxiety about the test and awaiting test results	179 (20.1)	129 (19.8)	50 (20.9)	0.802
Concerns about being able to understand test results	162 (16.9)	129 (17.5)	33 (15.0)	0.535
Opinions of family and friends	111 (13.9)	84 (13.7)	27 (14.5)	0.846

Table reports the frequency and weighted percentage of participants who indicated a factor would matter “a great deal” in their hypothetical decision about whether or not to have an MCD test. Participants could endorse multiple factors. The reported *p* values are from chi-square tests that compared participants who reported having a routine medical check-up within the last year to those who had not

^a^A total of 841 participants completed the decision-making survey activity on MCD testing

A higher percentage of participants who had received a routine medical check-up within the last year indicated doctor recommendation for getting the test as mattering a great deal in their MCD testing decisions compared to those who had not received routine medical check-up (54.2% vs. 28.1%, *p* < 0.001; Table 3). All other factors were comparable between the two groups (all other *p* > 0.05).

## Discussion

MCD testing is an emerging approach that has the potential to impact the current cancer screening and early detection landscape. In the current study, we examined perspectives on MCD testing among a national sample of adults in the US. Fewer than 10% of participants were aware of MCD testing prior to the survey, which likely reflects that MCD testing is not yet an approved/recommended screening strategy in the US. However, after receiving brief information about MCD testing, willingness to have an MCD test was high among participants. Indeed, about three-fourths of participants were willing to have an MCD test if it was free or covered by health insurance. This level of willingness is encouraging and is very similar to recent studies in both the US and the United Kingdom [15, 16, 19]. For example, two recent US studies also found that over 70% of participants were willing to have an MCD test [15, 16]. Our findings provide further evidence that a majority of adults in the US are interested in MCD testing. It is important to monitor temporal changes in willingness as research on MCD testing continues and, if MCD testing becomes approved/recommended, to then determine how these estimates of willingness translate into actual use of MCD testing.

Results of the current study highlight the importance of cost and health insurance coverage to MCD testing. Willingness to have an MCD test was much higher if the test was free than if it cost $1,000 out of pocket, which is similar to past research examining how out of pocket costs affect willingness to engage in new preventive health behaviors [25, 29]. Further, health insurance status was a strong correlate of willingness in multivariable analyses, and cost/insurance coverage was the most commonly endorsed factor that would matter in participants’ hypothetical MCD testing decisions. At the time of our study, the cost of two MCD tests that can detect a range of cancer types and that were already commercially available was about $1,000 each [11]. These costs are currently not covered by most health insurance plans, and our results suggest that if patients continue to be responsible for paying these costs out of pocket, it will likely lead to low utilization of MCD testing. If MCD testing becomes an approved/recommended screening approach, these costs could become covered for many individuals since the Affordable Care Act requires private insurers and other health insurance programs (e.g., Medicaid) to cover the costs of many preventive healthcare services, including recommended cancer screening tests [26]. Even if this coverage occurs, efforts will still be needed to ensure the accessibility of MCD testing among individuals who do not have health insurance and do not qualify for health insurance programs.

Similar to other cancer screening behaviors [30, 31], healthcare providers and healthcare utilization will play a central role in MCD testing. Indeed, nearly half of participants indicated doctor recommendation would matter in their MCD testing decisions, and having a recent routine medical check-up was correlated with willingness to have an MCD test in multivariable analyses. In recent studies, healthcare providers indicated multiple potential benefits of MCD testing for their patients (e.g., convenience, the tests are not intrusive, improved screening capabilities), as well as several concerns (e.g., lack of knowledge about MCD testing, strategies for how to educate and communicate with patients about MCD testing, ability to manage MCD test results, test performance) [32–34]. If MCD testing becomes approved/recommended, it will become increasingly necessary that healthcare providers are educated about this testing and provided with training on how to communicate with patients about MCD testing and test results. Education and communication training programs can improve both cognitive outcomes (e.g., knowledge, attitudes, and beliefs) among healthcare providers and the use of preventive healthcare services among their patients [35–38].

There are two additional important findings from the current study. First, multiple characteristics related to the performance of an MCD test (i.e., how well the test works, if test results indicate the potential site of cancer, the number of detectable cancer types) were among the mostly commonly endorsed factors that would matter in participants’ hypothetical MCD testing decisions. These characteristics align with the perceived benefits and response efficacy of MCD testing, which are key constructs in health behavior theories [39, 40]. Future efforts to educate patients about MCD testing should therefore feature content about these test performance characteristics. In doing so, the content should be guided by the current MCD testing landscape (e.g., tests vary on the number and types of detectable cancers) and inform patients about both the pros and cons (e.g., false positive results) of test performance. Second, willingness to have an MCD test was lower among participants who were non-Hispanic black or who had lower levels of education, which is noteworthy because mortality rates for many cancer types are higher among these communities [41]. The lower willingness for MCD testing may reflect that use of currently recommended cancer screening tests also tends to be lower among these same communities, which in turn is due to a range of factors including the social determinants of health [41]. If MCD testing becomes approved/recommended, it will be critical to monitor the use of MCD tests in order to identify and address any potential disparities in MCD testing across demographic characteristics. Such disparities in MCD testing could contribute to prolonging or even widening existing cancer-related disparities.

The study has several important strengths including a large sample size of participants from throughout the US and examining multiple topics related to MCD testing. Study limitations include the study’s cross-sectional design and lack of information on non-respondents. We recruited a convenience sample of participants from an online survey panel, though the data were weighted to represent the 45–80-year-old population of the US. This resulted in our study sample being very similar to the US population on several demographic and health-related characteristics [42, 43]. Members of this online panel complete surveys on a regular basis, which could have affected their responses. The study occurred prior to MCD testing being an approved/recommended screening approach and willingness to have an MCD test may overstate eventual behavior since intent does not always translate into health behavior [44]. The survey did not examine some aspects of MCD testing that may also affect people’s interest in and decisions about MCD testing (e.g., how frequently a person would need to have a test, what specific follow-up care would be needed after a positive MCD test, and the potential cost of follow-up care), as some of these details are not currently known.

Our study provides early insight into people’s willingness to have an MCD test, with results indicating that most adults in the US would be willing to have an MCD test. Our findings highlight the central roles that cost/insurance coverage, healthcare providers, and the performance characteristics of the MCD tests will likely play in people’s willingness and potential eventual use of MCD testing. Further, current willingness to have an MCD test differs by key demographic characteristics (i.e., race/ethnicity and socioeconomic status). Findings from our study can help guide the development and content of public health programs for patients and healthcare providers (e.g., education programs, communication training) that will become critical if MCD testing becomes an approved/recommended screening approach in the US.

## Data Availability

Deidentified data may be made available upon request.

## Code availability

Analyses were conducted using IBM SPSS version 29 (IBM Corp., Armonk, NY). Statistical code may be available upon request.
